# CT appearance of a patent impar umbilical artery in an adult woman and related anomalies: a case report and review of the literature

**DOI:** 10.1186/1757-1626-2-65

**Published:** 2009-01-20

**Authors:** Bernhard Glodny, Benjamin Henninger, Karin Hofmann, Thomas Trieb, Johannes Petersen, Peter Rehder

**Affiliations:** 1 Department of Radiology, Innsbruck Medical University, Anichstrasse 35, 6020, Innsbruck, Austria; 2 Neurourology, Department of Neurology, Innsbruck Medical University, Innsbruck, Austria

## Abstract

**Background:**

We report on a case of an impar umbilical artery (IUA) in an 18-year-old woman.

**Case presentation:**

The aorta branched off at level L2 into a ventral IUA and a dorsal aorta. The strong IUA produced the inferior mesenteric artery (IMA), the renal artery of a left-sided duplex kidney, and the right-sided ovarian artery before it turned to the right to merge into the right common iliac artery. From the aorta arose the lumbar arteries, the median sacral artery, lateral sacral arteries, and iliolumbar arteries before it turned to the left. Both vessels were connected by an artery 0.8 cm in diameter running infraperitoneal, from the left side of which the uterine artery arose for a left paramedian uterus didelphys.

**Conclusion:**

This anatomical situation is presented for the first time using an arterial contrast enhanced CT and is discussed within the context of previously known cases.

## Background

Persistent, patent, single aberrant abdominal umbilical arteries (SUA) are extremely rare [1]. The variants, also known as impar umbilical artery (IUA) are classified in three forms according to their origin: the proximal branching site of the IUA from the aorta at level L1 or L2, the more distal branching of the IUA at level L3 to L5, and the branching site from the superior mesenteric artery (SMA). The IUA is usually associated with sirenomelia or caudal regression [2]. We are reporting here on the unusual case of an 18-year-old patient with a type I right IUA with no accompanying symmelia. A strong infraperitoneal connection between the right IUA and the left-sided aorta, described here for the first time, is discussed in the context of the previously known cases. This is the first presentation of an IUA using computed tomography.

## Case presentation

The 18-year-old patient with terminal renal insufficiency was presented in 2001 for a contrast medium enhanced CT scan of the abdomen to clarify a malformation syndrome during preparations for the transplant. As a young child, she had undergone a mixed augmentation of the bladder due to congenital agenesis of the bladder and urethra. The ureter of a left-sided iliolumbar incomplete duplex kidney with duplex pelvis and bifid ureter was implanted in the pouch. In the further course, the patient suffered recurrent urinary tract infections and pain in the lower right abdomen, in the course of which renal function continued to deteriorate. At this time, only a grade two pulmonary valve stenosis and renal agenesis on the right side had been diagnosed. The CT scan (General Electric Light Speed QX/i, 2.5 mm Collimation, 120 ml Ultravist 370, Schering, Berlin, Germany), and subsequent MRI (Siemens Avanto 1.5 T, Siemens, Erlangen, Germany) showed complex anomalies. The CT localizer showed right hip dysplasia with beginning shepherd's crook deformity. The CT also showed a slight caudal regression with hypoplasia of the coccyx and the sacral vertebrae 4 and 5. The spinal canal between S1 and S2 was widened. In the left iliolumbar region there was a 10.2 cm long duplex kidney with two separate renal pelves and a bifid ureter that was joined distally. Both branches of the ureter were widened to a diameter of 1 cm and the patient had grade 1 hydronephrosis. There was a 1.6 cm large concrement in the "neobladder". The MRI led to the diagnosis of uterus didelphys, but with only one left and one right ovary.

**Figure 1:**
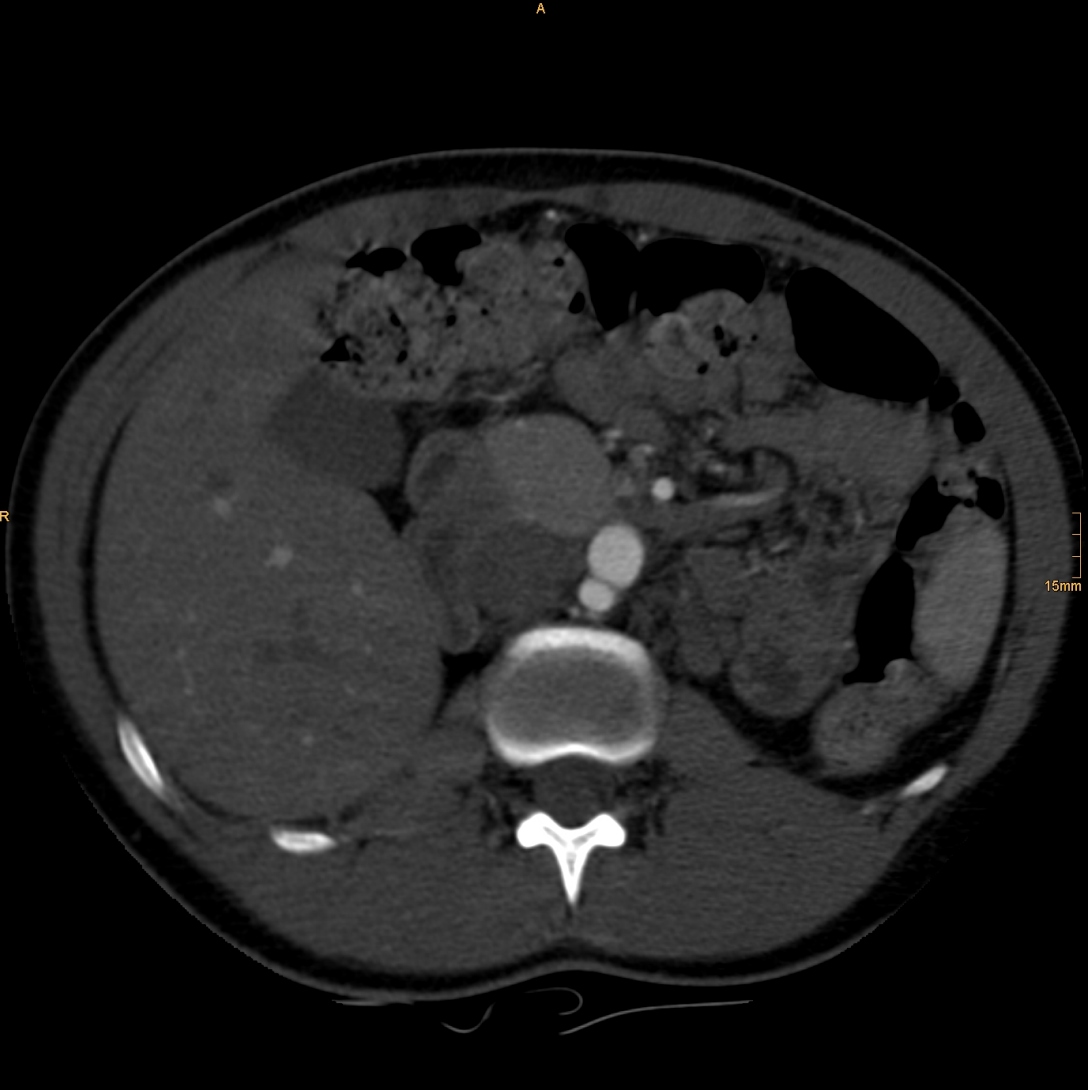
**The aorta, dividing into a 15 mm diameter ventral branch and an 8 mm diameter dorsal branch at level L1/2**.

**Figure 2:**
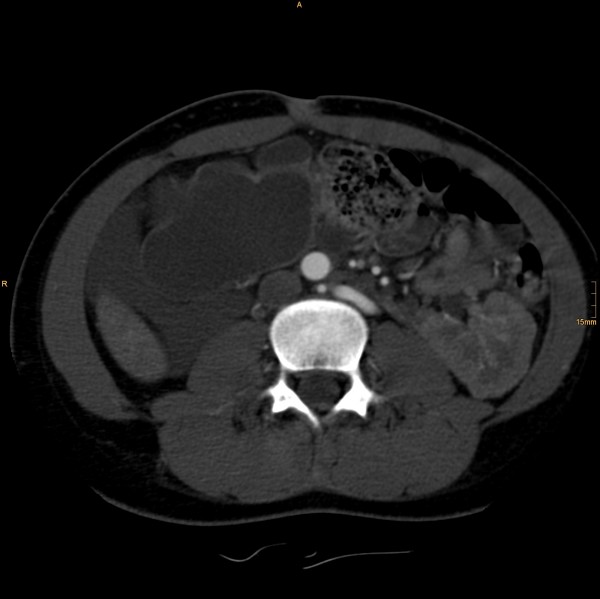
**The ventral branch dividing into a 4 mm wide medial sacral artery and a 6 mm wide stem proceeding to the left side**.

**Figure 3:**
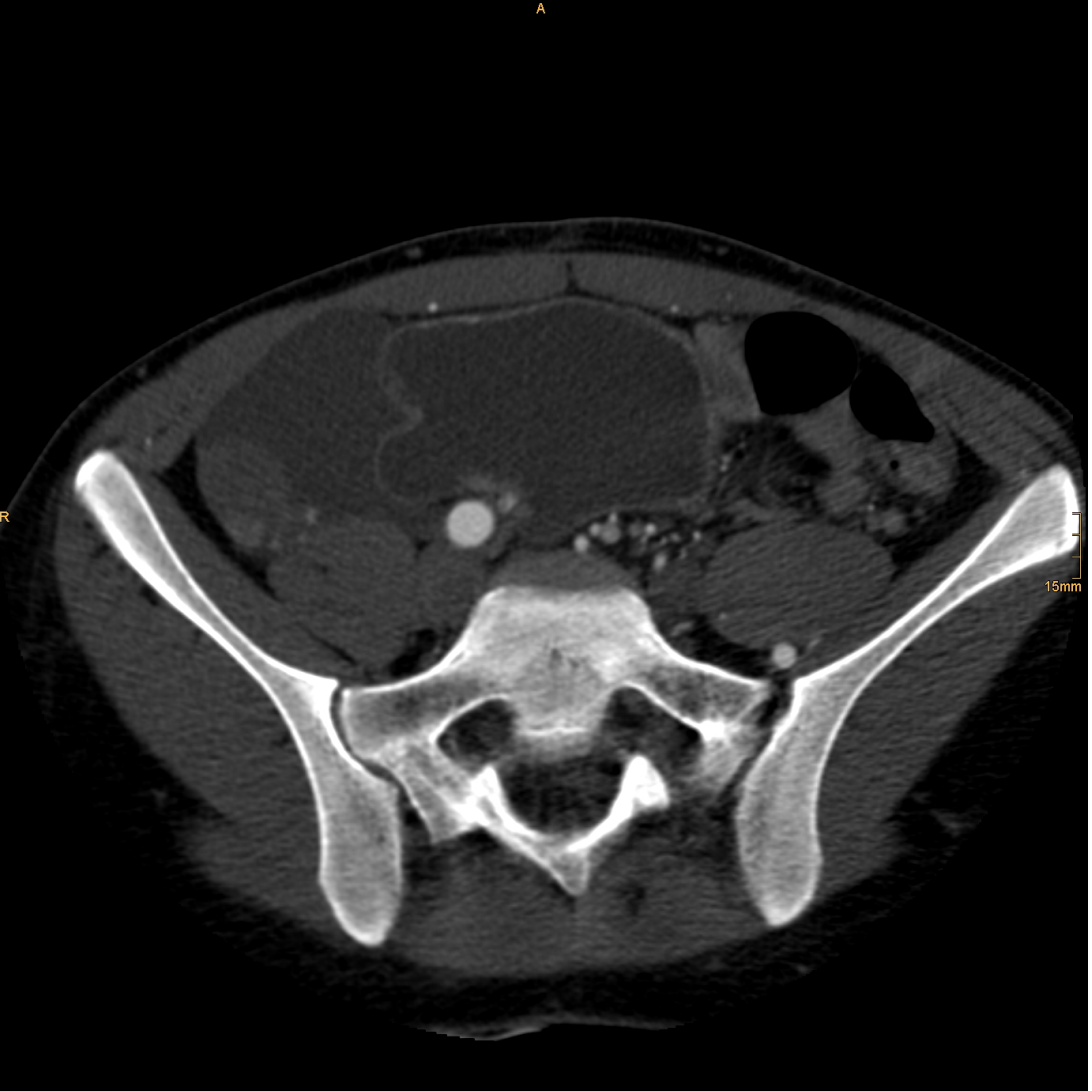
**Then taking a position dorsal to the left psoas muscle**.

**Figure 4:**
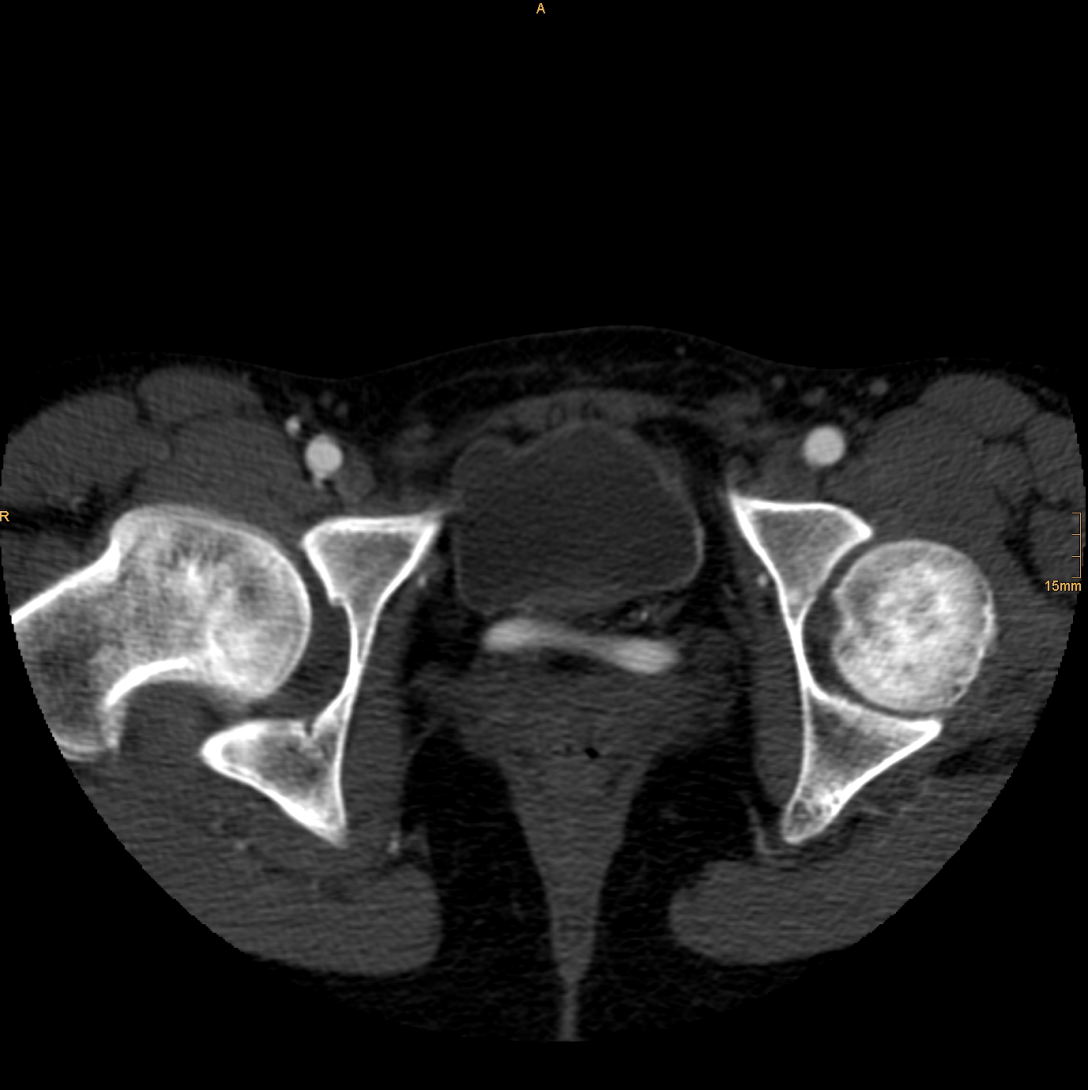
**Then anastomizing with an 8.5 mm diameter artery coming from the right and crossing over infraperitoneal**. It continued a normal course as the left common femoral artery.

The following vascular situation was seen: the aorta produced a first lumbar artery at level L1 before dividing into a 15 mm diameter ventral branch and an 8 mm diameter dorsal branch at level of the disc L1/2 (Figure 1). The dorsal branch, considered the aorta, first produced a common stem of a second lumbar artery before dividing into a 4 mm wide median sacral artery and a 6 mm wide branch proceeding to the left side (Figure 2). The third and fourth right lumbar arteries originated from the medial sacral artery; the third and fourth left lumbar arteries from the branch described proceeding to the left side. The latter branch took a position dorsal to the left psoas muscle, where it still had a diameter of 6 mm, producing the superior gluteal artery (Figure 3), inferior gluteal artery, and left obturator artery in that order, and anastomized immediately thereafter at the level of the femoral head with an 8.5 mm diameter artery coming from the right and crossing over infraperitoneal (Figure 4). As its diameter increased to 9 mm, it continued a normal course as the left common femoral artery.

**Figure 5:**
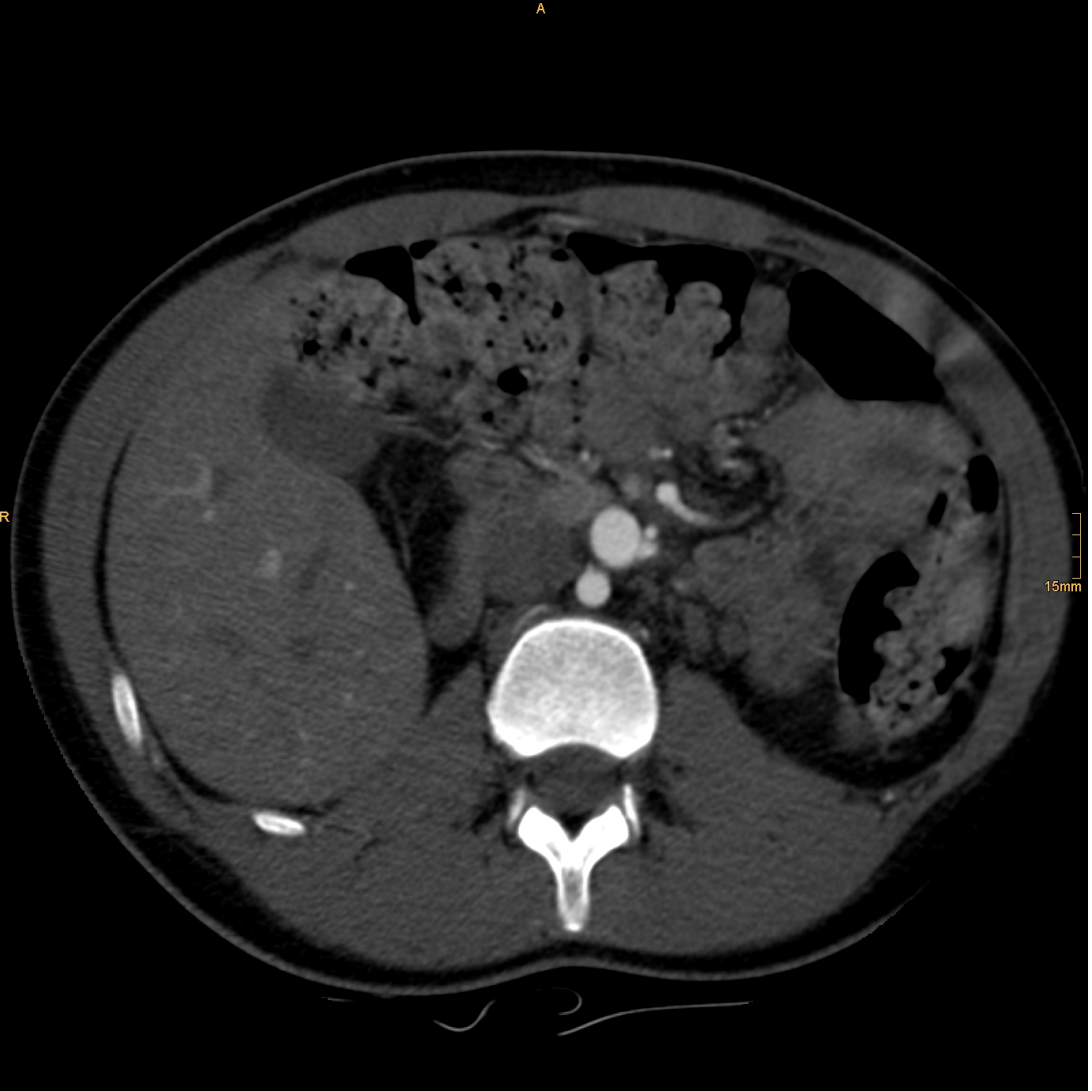
**The ostia of the left renal artery immediately dorsal to that of the inferior mesenteric artery (IMA), branching off the IUA**.

**Figure 6:**
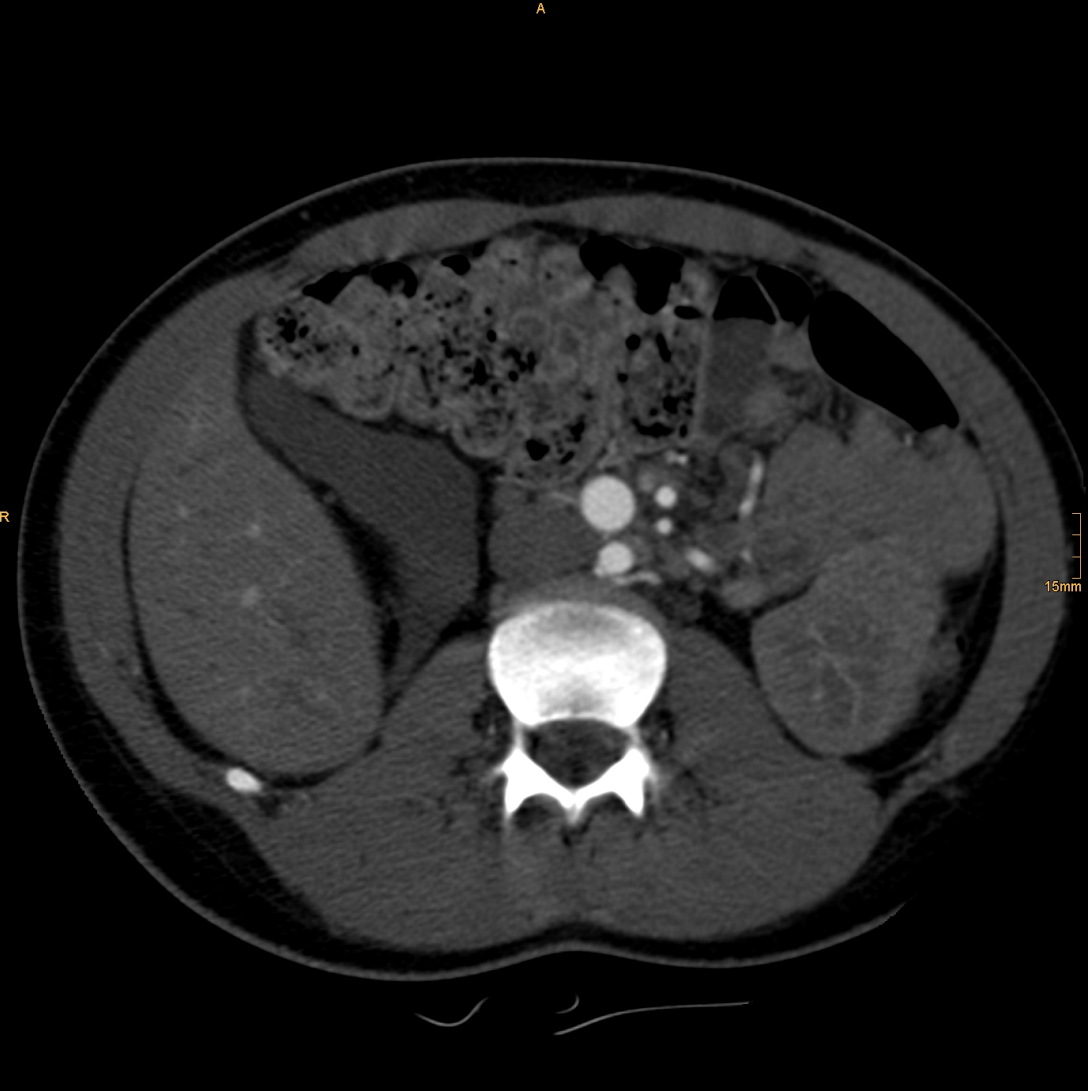
**The ostium of the right ovarian artery, branching off the IUA**.

**Figure 7:**
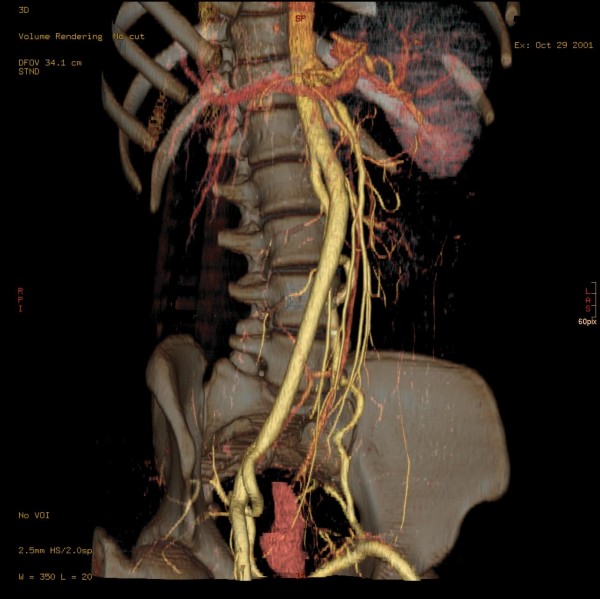
**Approximately 70° LAO rotated semitransparent volume rendering reconstruction view of the partially wavy left aorta**.

**Figure 8:**
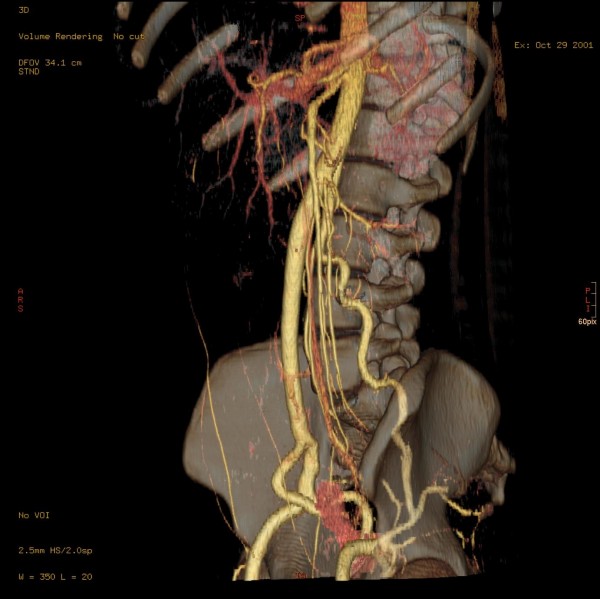
**Approximately 70° RAO rotated semitransparent volume rendering reconstruction view of the impar umbilical artery, including the branch of the uterine artery**.

**Figure 9:**
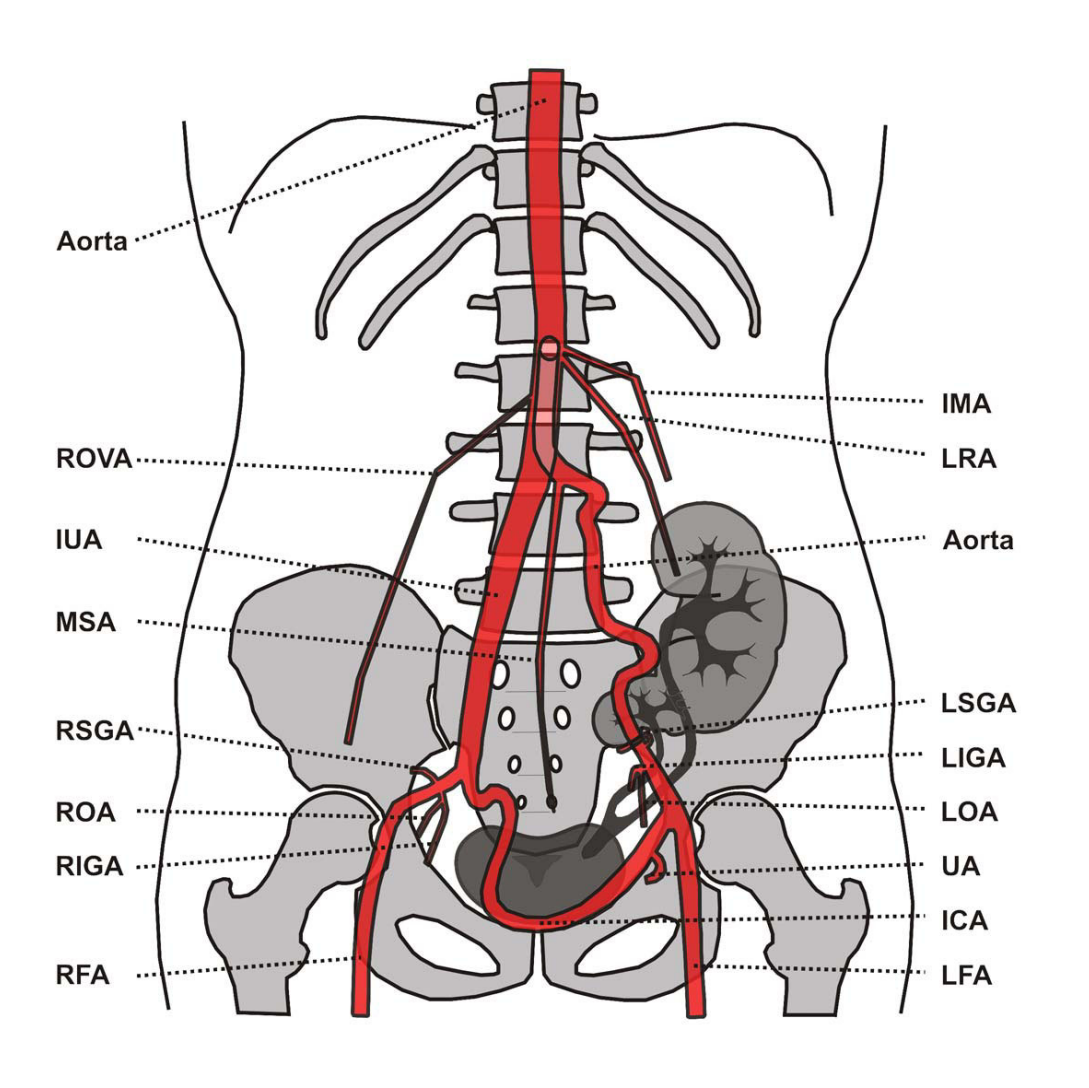
**Shows a schematic rendering of the vascular situation, drawn from a series of volume rendering, and maximum intensity projection reconstructions of the CT and MRI images**. ICA – Inferior communicating artery; IMA – Inferior Mesenteriv Artery; IUA – Impar Umbilica Artery; LFA – Left Femoral Artery; LOA – Left Obturatory Artery; LIGA – Left Inferior Gluteal Artery; LRA – Left Renal Artery; LSGA – Left Superior Gluteal Artery; MSA – Median Sacral Artery; RFA – Right Femoral Artery; RIGA – Right Inferior Gluteal Artery; ROA – Right Obturatory Artery; ROVA – Right Ovarian Artery; RSGA – Right Superior Gluteal Artery; UA – Uterine Artery.

The thicker ventral "branch" of the aorta, the IUA, produced the inferior mesenteric artery and the left renal artery at the level of mid-L2, approx. 11 mm distal to the bifurcation. The ostia of the left renal artery was immediately dorsal to that of the inferior mesenteric artery (IMA) (Figure 5), while the left renal vein crossed over to the right side dorsal to the impar umbilical artery and ventral to the aorta. Somewhat further caudal, still at the level of L2, the right ovarian artery branched off (Figure 6), before dividing into a short right common iliac artery and the already mentioned vessel crossing over infraperitoneal to the left side. The latter crossed the mid-line without producing any branches and produced a strong uterine artery only shortly before the anastomosis with the aorta on the left side. Figure 7 shows an approx. 70° LAO rotated semitransparent volume rendering reconstruction view of the partially wavy left aorta, Figure 8 shows an approx. 70° RAO rotated semitransparent volume rendering reconstruction view of the impar umbilical artery, including the branch of the uterie artery. Figure 9 shows a schematic rendering of the vascular situation, drawn from the volume rendering reconstructions of the CT and MRI images. Shortly thereafter, the patient underwent a kidney transplant into the small right pelvis, in which the renal artery was anastomized end-to-side to the distal segment of the impar umbilical artery. Aside from several urinary tract infections requiring antibiotic therapy, the further course was uncomplicated with good function of the transplant.

## Discussion

Beginning on the thirteenth day of development, the embryo is connected to the placenta [3]. By the end of the fifth week, the umbilical cord, which is formed from portions of the body stalk, the primitive yolk sac, and the amnion, contains the Vitelline duct with its artery and vein, the allantois, two umbilical arteries, and a vein [4]. The data on the incidence of variations of the single umbilical artery (SUA) fluctuate greatly depending on the parity of the mother, origin, the umbilical cord segment examined, and the examination method used [3]. The incidence lies between 0.3 and 0.5% for healthy newborns [5,6]. The SUA can be divided into four types according to its etiology [7], all of which can be associated with other fetal malformations and fetal chromosomal abnormalities, intrauterine growth retardation, prematurity, and placental abnormalities [3]. The situation presented here of a persistent, patent, single aberrant abdominal umbilical artery, or IUA is interesting for various reasons. It is a very rare case of a SUA, which could be confused at first glance with the considerably more common variation of a high bifurcation of the aorta at level L2 [8]. However, the higher bifurcation site of the aorta in the case presented here and the formation of parietal lateral branches exclusively from the dorsal vessel on the left side contradict this view. On the other hand there is no indication that the stronger ventral vessel turning toward the right side producing only visceral branches could be a second "aorta". A closer look at the two cases of a "double" abdominal aorta described in literature shows them to be well-documented cases of most likely arteriosclerotic aortic dissection [9,10].

The type I IUA is normally associated with such severe defects that it is usually described only in a fetus [1,11,12]. It is closely associated as part of a caudal regression – sirenomelia spectrum, which is probably caused by an embryonic deficiency of the caudal mesoderm [1]. All of the cases in Senior's overview displayed an imperforate anus and an undifferentiated cloaca. Most of them were also sympodial [2]. By contrast, our patient had agenesis of the bladder, but no symmelia. Due to the combination of bladder and urethral agenesis, right renal agenesis, uterus didelphys, the hypoplastic distal segment of the sacrum, the hypoplastic coccyx, and the right hip dysplasia, we must assume an abortive caudal regression in her case as well. She seems to be the only patient known to have reached adulthood with this combination of anomalies. The right hip dysplasia could be viewed as simply a sirenoid malformation. According to this view, it would be an abortive form of a left-sided monopus [13], possibly caused by a deficient supply of the lower right extremity due to the loss of blood to the strong vessel running infraperitoneal to the left side.

This strong infraperitoneal connection between the right-sided IUA and the left-sided aorta is unique thus far in literature. The fact that the long, meandering right-sided segment of this vessel does not produce any branches after the common iliac artery branches off, which was defined as such due to its subsequent branching pattern into the internal iliac artery and common iliac artery, causes us to assume that it is the continuation of the IUA itself. The branching off of the strong uterine artery on the left side, somewhat proximal of the anastomosis with the distal segment of the aorta leads us to believe that the left side is embryonically most likely a short segment of the left internal iliac artery. The segment of the aorta anastomized to the infraperitoneal vessel was ultimately defined as the external iliac artery due to the branching off of the left superior and inferior gluteal arteries and the left obturator artery. In any case, from where and to where the left-sided common, external, and internal iliac arteries and the left inferior gluteal artery are represented in the anastomosis area is unresolved. And the question of whether the umbilical artery on the left side is also represented in the course of the infraperitoneal vessel and if so, where precisely [2] also remains speculation. It should be mentioned only that the pattern of the origin and course of the vessels, aside from the aorto-iliacal/umbilical anastomosis, shows a detailed correspondence with the case of a siren described by Odisio in 1892 [14]: the strong IUA supplies blood to both sides through a vessel crossing infraperitoneal over to the left side, interpreted as the right common iliac artery.

The malformation pattern of the IUA, that was associated in this case with caudal regression and an abortive form of a left-sided monopus, can also be seen in adulthood and should be recognized as such. Since the prognosis of this malformation is not always unfavorable, the task of diagnosing this situation may also be up to a radiologist. Due to the complexity of the vascular situation and associated malformations, imaging methods must be chosen individually for every case. Due to the good visualization of vessels in relation to the overall anatomical situation, CT angiography is probably the method of choice, combined with targeted MRI for the clarification of unexpected accompanying malformations of the genital and internal organs, kidneys, and lower urinary tract.

## Abbreviations

IUA: impar umbilical artery; IMA: inferior mesenteric artery; SUA: single umbilical artery; SMA: superior mesenteric artery

## Consent

Written informed consent was obtained from the patient for publication of this case report and accompanying images. A copy of the written consent is available for review by the Editor-in-Chief of this journal.

## Competing interests

The authors declare that they have no competing interests.

## Authors' contributions

BG was the primary person responsible for the writing of the manuscript. BH, KH, TT, JP and PR edit and coordinated the manuscript. All authors read and approved the final manuscript.
